# Feasibility test of WORK-ON: a vocational rehabilitation intervention for people with chronic inflammatory arthritis

**DOI:** 10.1186/s12891-024-07862-8

**Published:** 2024-10-01

**Authors:** Christina Merete Tvede Madsen, Jeanette Reffstrup Christensen, Ann Bremander, Linda Eggen, Jette Primdahl

**Affiliations:** 1 The Danish Center for Expertise in Rheumatology, Danish Hospital for Rheumatic Diseases, University Hospital of Southern Denmark, Engelshøjgade 9A, Sønderborg, 6400 Denmark; 2 Department of Regional Health Research, University of Southern Denmark, Odense, Denmark; 3 Research Unit of General Practice, Department of Public Health, University of Southern Denmark, Odense, Denmark; 4 DRIVEN—Danish Centre for Motivational and Behavior Science, Department of Sports Science and Clinical Biomechanics, University of Southern Denmark, Odense, Denmark; 5 Research Unit of General Practice, Aarhus, Denmark; 6 Section of Rheumatology, Department of Clinical Sciences Lund, Lund University, Lund, Sweden; 7 Hospital Sønderjylland, University Hospital of Southern Denmark, Aabenraa, Denmark

**Keywords:** Axial spondyloarthritis, Medical research council, Psoriatic arthritis, Presenteeism, Rheumatoid arthritis, Sick leave, Work ability, Work rehabilitation

## Abstract

**Background:**

People with chronic inflammatory arthritis (IA) often have a reduced work ability. Consequently, they are at high risk of losing their jobs and being permanently excluded from the labor market. Therefore, we developed a new context-specific vocational rehabilitation intervention for people with IA based on the Medical Research Council’s framework for complex interventions. This intervention is called “WORK-ON” and consists of: (1) Initial assessment and goal setting by an occupational therapist experienced in rheumatology rehabilitation; (2) coordinated support from the same occupational therapist, including assistance in navigating the primary and secondary healthcare and social care systems; (3) group sessions for peer support; and (4) individually tailored consultations with physiotherapists, nurses, and/or social workers. This study investigates the feasibility of WORK-ON.

**Methods:**

A 6-month single-arm feasibility study with a pre-test post-test design was conducted to evaluate recruitment, intervention fidelity and delivery, data collection, and possible outcome measures. Work ability was the primary outcome, and sick leave, quality of life, fatigue, pain, physical activity, sleep, and well-being were the secondary outcomes evaluated.

**Results:**

In total, 19 participants (17 women and 2 men) with a median age of 55 years (range, 34–64) participated and completed WORK-ON. Of these, 17 participants completed patient-reported outcomes at baseline and follow-up, and the results indicated a tendency to improvement in work ability, quality of life, level of physical activity, decrease in pain, and increase in days of sick leave during the 6-month intervention period. The rehabilitation clinicians spent an average of 15.3 h per participant, and the participants spent an average of 13.5 h in the intervention.

**Conclusions:**

WORK-ON is considered feasible and has the potential to increase work ability among people with IA who are concerned about their future ability to keep working. Though, an adjustment of the intervention is needed before testing in a randomized controlled trial.

## Background

Impaired work ability is common among people with chronic inflammatory arthritis (IA), which includes rheumatoid arthritis (RA), axial spondyloarthritis (axSpA), and psoriatic arthritis (PsA). Approximately 30–40% of people with IA do not tolerate or derive sufficient benefits from pharmacological treatment [1–3]. People with IA often experience functional impairments, anxiety, depression, and disturbed sleep, and more than 50% of patients experience substantial fatigue and pain [1, 4–6]. Consequently, they have a high risk of losing their jobs and being permanently excluded from the labor market [7–10]. Up to 40% of those with IA lose their jobs within the first few years of being diagnosed, and they find it difficult to return to work [7, 8, 11–15]. Therefore, early support is crucial to enable people with IA to continue in paid employment. Challenging aspects of work, such as physically demanding tasks, stationary work, or varied working hours, can lead to increased sick leave among people with IA, compared with the general population [16, 17]. The direct and indirect costs of IA—primarily, the expensive treatments required and patients’ reduced capacities for work—are substantial, affecting both individuals and societies [11, 18].

Participation in paid work is important for an individual’s sense of identity and belonging; their self-esteem, everyday routine, and social relationships; and their ability to fulfil societal expectations [19–24]. Vocational rehabilitation (VR) is often complex and involves various professionals and interventions [18]. Few studies have described the effects of VR on people with IA, but our previous systematic review showed VR can affect work ability, sick leave, and job loss among people with IA [18]. Using the Medical Research Council’s (MRC) framework for developing and testing complex interventions [25], we developed a VR intervention called “WORK-ON” [26]. The aim of WORK-ON is to increase work ability and decrease sick leave and job loss in the longer term. WORK-ON is based on evidence from our previous systematic review [18], interviews with people with IA who consider themselves at risk of losing their job [16], and input from rehabilitation clinicians (RCs) and employers [26, 27]. We included in the intervention occupational balance [28], self-management [29], shared decision-making (SDM) [30], and Focused Acceptance and Commitment Therapy (FACT) [31] as key theories and approaches. Further, a logic model was developed to highlight the relationships between activities and expected outcomes, and outcome measures were chosen based on this information [26].

The MRC framework recommends using a feasibility test to evaluate eligibility criteria, recruitment, outcomes, fidelity, and delivery of interventions prior to conducting a randomized controlled trial (RCT) [25]. Therefore, this study sought to evaluate the feasibility of WORK-ON, focusing on the recruitment procedure, intervention fidelity and delivery, data collection, possible outcome measures, and the optimal primary outcome measure for a subsequent RCT.

## Methods

### Design and setting

We performed a single-arm 6-month feasibility study at the Danish Hospital for Rheumatic Diseases (DHRD; Soenderborg, Denmark). Participants were recruited from the outpatient department at the DHRD. The Regional Committees on Health Ethics for Southern Denmark stated no formal ethical approval was necessary for this study (journal number, 20192000-105). Findings were reported in accordance with the Consolidated Standards of Reporting Trials (CONSORT) statement for pilot and feasibility studies [32].

### Intervention content

**Fig. 1:**
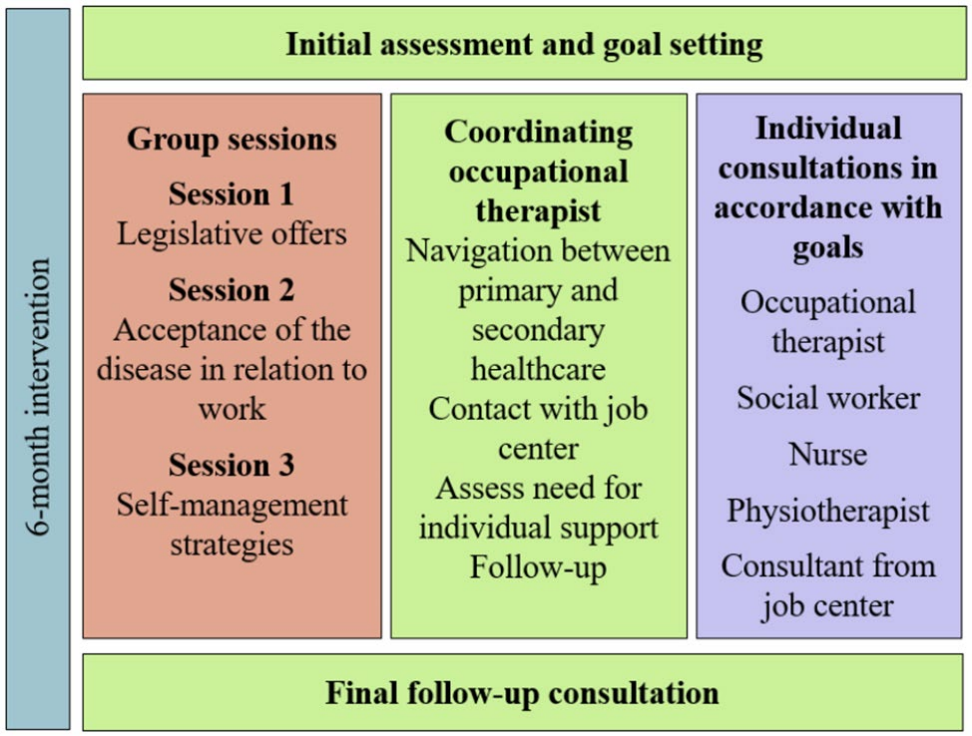
Content of the 6-month WORK-ON intervention [26]

WORK-ON was implemented over a period of 6 months and was planned to include 6 to 18 consultations (approximately 14–21 h), including group sessions, depending on participant needs. WORK-ON included the following four elements:

1. Initial assessment by an occupational therapist (OT) experienced in rheumatology rehabilitation using the Work Experience Survey for Patients with Rheumatic Conditions (WES-RC) [33], followed by setting goals and planning actions based on SDM [30].
2. Coordination and support of individual self-management by the same OT throughout the VR to achieve occupational balance in everyday life [28, 29]. This included support in navigating the primary and secondary healthcare and social care systems, as well as access to the municipal job center. The coordinating OT could schedule a minimum of three consultations with each participant.
3. Three group sessions for education and peer support: one session with a social worker focusing on legislation, one with a nurse focusing on acceptance of the disease in relation to work, and one with an OT focusing on coping strategies. The group sessions were followed by a consultation with the coordinating OT to determine whether the participant needed individual consultations with other RCs at the DHRD.
4. Optional individually tailored VR consultations: a maximum of two consultations per RC, depending on the participants’ needs and goals. These included consultations with physiotherapists to recommend individually tailored physical activities and exercise; with nurses to better understand the disease and how to manage fatigue and pain; with social workers to understand legislation; and with OTs to recommend hand exercises, small aids, and supervision in ergonomic positions (Fig. 1).

Other details of the development, content, and timeline of the intervention are provided elsewhere [26].

### Eligibility criteria

The inclusion criteria were:

1. age ≥ 18 years.
2. diagnosed with RA, axSpA, or PsA.
3. in paid work (full-time or part-time, self-employed) or full-time education
4. if on sick leave, had to have been for less than four weeks.
5. able to read and understand Danish.
6. willing to participate in group sessions.
7. answered “unlikely” or “not certain” to question 6 from the Work Ability Index (WAI) questionnaire: *“*Do you believe, according to your present state of health, that you will be able to do your current job two years from now*?”* [34].

The exclusion criteria were:

1. not able to attend WORK-ON because of cognitive issues.
2. having / will be having changes in the pharmacological treatment expected to affect work ability.
3. major surgery within the past six months or planned surgery.
4. under investigation for other severe comorbidities.
5. problems retaining work due to comorbidities other than IA, such as psychiatric illness, heart disease, cancer, or chronic obstructive pulmonary disease.
6. undergoing / due to undergo other rehabilitation in the hospital or municipality.

### Recruitment procedure

All outpatients with IA completed questionnaires for the national quality database DANBIO using a touchscreen or computer before each outpatient consultation at the DHRD [35]. For patients with RA, axSpA, or PsA aged ≥ 18 years, the regular questionnaires were followed by an extra question asking whether they were currently in paid employment. If they were, then question #6 from the WAI questionnaire was asked: *“*Do you believe, according to your present state of health, that you will be able to do your current job two years from now*?”* [34]. Patients who answered “unlikely” or “not certain” were encouraged to insert their phone number if they were interested in hearing about a new offer of support for maintaining employment: WORK-ON.

The first author (CTM) contacted the interested patients by phone and screened them using the eligibility criteria. Eligible patients were offered further information about the study and written participant and consent information was sent to them by e-mail. If a patient agreed to participate, their written consent was collected before the first WORK-ON consultation. A baseline questionnaire was sent to each participant’s e-mail address using Research Electronic Data Capture (REDCap). REDCap is a secure web application for building and managing online surveys. Sociodemographic and disease-related information was collected in addition to the completed questionnaires.

### Intervention delivery

The RCs who delivered WORK-ON received 8 h of training prior to commencing the feasibility study. This included information on the background, content, development, and design of WORK-ON, as well as on procedures for the group sessions, individual consultations, booking, and attendance registration. Furthermore, the RCs, which practice a person-centered approach at the DHRD, also received training in FACT [31] during four 2-hour modules at the start of the feasibility study. A person-centered approach and FACT are important elements of WORK-ON. The training was delivered by members of the research team (CTM and JP) and a psychologist. In addition, the coordinating OTs received 3 h of training in using the WES-RC [33].

### Intervention fidelity

To study fidelity, journal entries were read to assess whether the intervention was delivered as described in the manual. Furthermore, for each participant, the RCs completed a logbook with the following information: date, type of consultation, time required during and after the consultation, materials offered to the participant (e.g., pamphlets), referrals to other RCs, and professional skills used, as well as ideas to improve the manual and any successes or challenges experienced in each consultation. There were learning meetings throughout the intervention period with the RCs and CTM. To conduct a process evaluation, two focus group interviews with the RCs, along with semi-structured interviews with the participants completing WORK-ON, were planned. The results from the process evaluation will be reported elsewhere.

### Outcome measures

Demographic information (e.g., age, sex, diagnosis, year of diagnosis, and marital status) was collected at baseline. Patient-reported outcome measures were recorded at baseline and at the end of the intervention (6 months later).

Two possible primary outcome measures (the WAI single item and the Work Productivity and Activity Impairment Questionnaire: General Health [WPAI: GH]) were tested in the feasibility study to determine which to use as the primary outcome in a subsequent RCT (Table 1). Following the MRC framework, outcome measures were selected using the available evidence, the underlying intervention theories, and the logic model [25]. Information on employment status (e.g., in full-time work, part-time work, self-employed, flexi-job, or in full-time education) was collected at baseline and at the end of the 6-month intervention.

**Table 1:** Outcome measures

Outcome	Measurement	Description	Reliability and validity
***Primary outcome***			
Work ability	WAI single item [34]	A visual analogue scale to compare perceived work ability with lifetime best score ranging from 0 “completely unable to work” to 10 “work ability at its best”)	Good reliability and validity [36]
	WPAI: GH [37]	Six questions to measure absenteeism, presenteeism, and the effect of health problems on the participants’ work ability and performing of regular activities during the previous 7 days. Expressed as time impaired (%). Higher numbers indicate greater impairment and less productivity due to health problems	Good reliability and validity [37]
***Secondary outcomes***			
*Outcome*	*Measurement*	*Description*	
Sick leave due to IA	Reported each month during the intervention period via text message reminders	Reported as number of days	Not applicable
Occupational balance	OBQ-11 [38, 39]	Measures experience of balance in terms of the amount and variation of everyday activities. Eleven items and four response levels (0 = completely disagree, 1 = tend to disagree, 2 = tend to agree, and 3 = completely agree). Higher scores are better	Translated but not validated in Danish. A Swedish study concluded OBQ-11 has good reliability [38, 39]
Health-related quality of life	EQ-5D-5 L [40]	Generic measure to assess population health-related quality of life (mobility, self-care, usual activities, pain/discomfort, and anxiety/depression) with five response levels (1 = no problems, 2 = slight problems, 3 = moderate problems, 4 = severe problems, and 5 = extreme problems) and a visual analogue scale (EQ-VAS, 0–100, 100 is best) reporting the participant’s self-rated health	Moderate to strong reliability and validity [40]
Pain	Measured by asking about pain experienced over the previous 4 weeks and the extent to which physical pain affected work and household chores [41]	Five response levels (1 = no pain, 2 = slight pain, 3 = moderate pain, 4 = severe pain, and 5 = extreme pain)	Not reported*
Fatigue	BRAF-NRSv2 [42]	Three numerical rating scales (0–10: 10 is the best score): fatigue level (severity), effect on life (impact), and coping, anchored by ‘no fatigue’ and ‘totally exhausted’, ‘no effect’ and ‘a great deal of effect’, and ‘very well’ and ‘not at all well’, respectively	Valid and reliable for use in a Danish setting [42]
Physical activity	“Over the previous year, how would you describe your physical activity?” “How many days a week are you physically active for at least 30 minutes?” “On a typical workday, how much sitting time do you have in the following situations?” (41)	Four response levels ([1] hard exercise; [2] sports, heavy gardening, etc.; [3] walking, cycling, etc.; and [4] seated activities) Eight response levels (0 days, 1 day, 2 days, 3 days, 4 days, 5 days, 6 days, 7 days) Reported as hours and minutes using transport, seated at work/school/education, leisure time using a screen, and other leisure time (meals, reading, etc.)	Not reported*
Well-being	WHO-5 Well-Being Index [43]	Short generic rating scale measuring subjective mental well-being the past 14 days ([1] “I have felt cheerful and in good spirits,” [2] “I have felt calm and relaxed,” [3] “I have felt active and vigorous,” [4] “I woke up feeling fresh and rested,” and [5] “My daily life has been filled with things that interest me.”) Each item is scored from 5 (all the time) to 0 (none of the time). The raw score ranges from 0 to 25 and is multiplied by 4 to give a final score from 0 (worst imaginable well-being) to 100 (best imaginable well-being)	Adequate validity [43]
Sleep	“How many hours and minutes did you sleep in a normal night during the previous 4 weeks?” “During the previous 4 weeks, did you get enough sleep to feel rested?” Questions about how you sleep: “Did you have trouble falling asleep?” “Did you wake up several times at night and have difficulty falling asleep?” “Did you wake up early and were unable to fall asleep again?” “Did you sleep restlessly?” [41]	Reported as hours and minutes Three response levels (1 = yes; 2 = yes, but not often enough; and 3 = no, never) Four response levels (1 = not in the last four weeks, 2 = less than once a week, 3 = 1–2 times a week, and 4 = three or more times a week)	Not reported*

BRAF-NRSv2, Bristol Rheumatoid Arthritis Fatigue Numerical Rating Scales version 2; EQ-5D-5 L, European Quality of Life-5 Levels; EQ-VAS, EuroQol Visual Analogue Scale; IA, inflammatory arthritis; OBQ-11, Occupational Balance Questionnaire; WAI, Work Ability Index; WHO, World Health Organization; WPAI: GH, Work Productivity and Activity Impairment Questionnaire: General Health; *, from a Danish national health profile questionnaire ‘How are you?’ [41]

Cognitive interviewing was used to test the readability, explanatory text, and response options of the outcome measures prior to the feasibility test [44]. Three patients who fulfilled the study inclusion criteria were asked to complete the questionnaires with the first author present. While completing the questionnaires, the patients were encouraged to comment if something was unclear or imprecise and on whether the number of questions was acceptable [44]. These interviews did not lead to any changes in the choice of patient-reported outcome measures.

### Data analysis

Data from the 17 participants who completed baseline and 6-month follow-up assessments were included in the analysis, as recommended by CONSORT [32]. The patient-reported outcome measures were scored in accordance with the developers’ recommendations. Descriptive statistics were used to describe participants’ characteristics. Medians and interquartile ranges were used for continuous data because of the small sample size. Frequencies and percentages were used for categorical data. The mean was used to report sick leave so these data could be compared with data from Statistics Denmark. The questions about pain and sleep were from a Danish National Health Profile and had no scoring instructions [41]. For the questions about pain, two categories were merged (“none” and “slight”) and for questions about sleep, two categories were merged (“less than once a week” and “1–2 times a week”) because of the low number of participants. Statistical analyses were performed using Stata software (ver. 17; StataCorp, College Station, TX, USA).

## Results

### Recruitment and inclusion

**Fig. 2:**
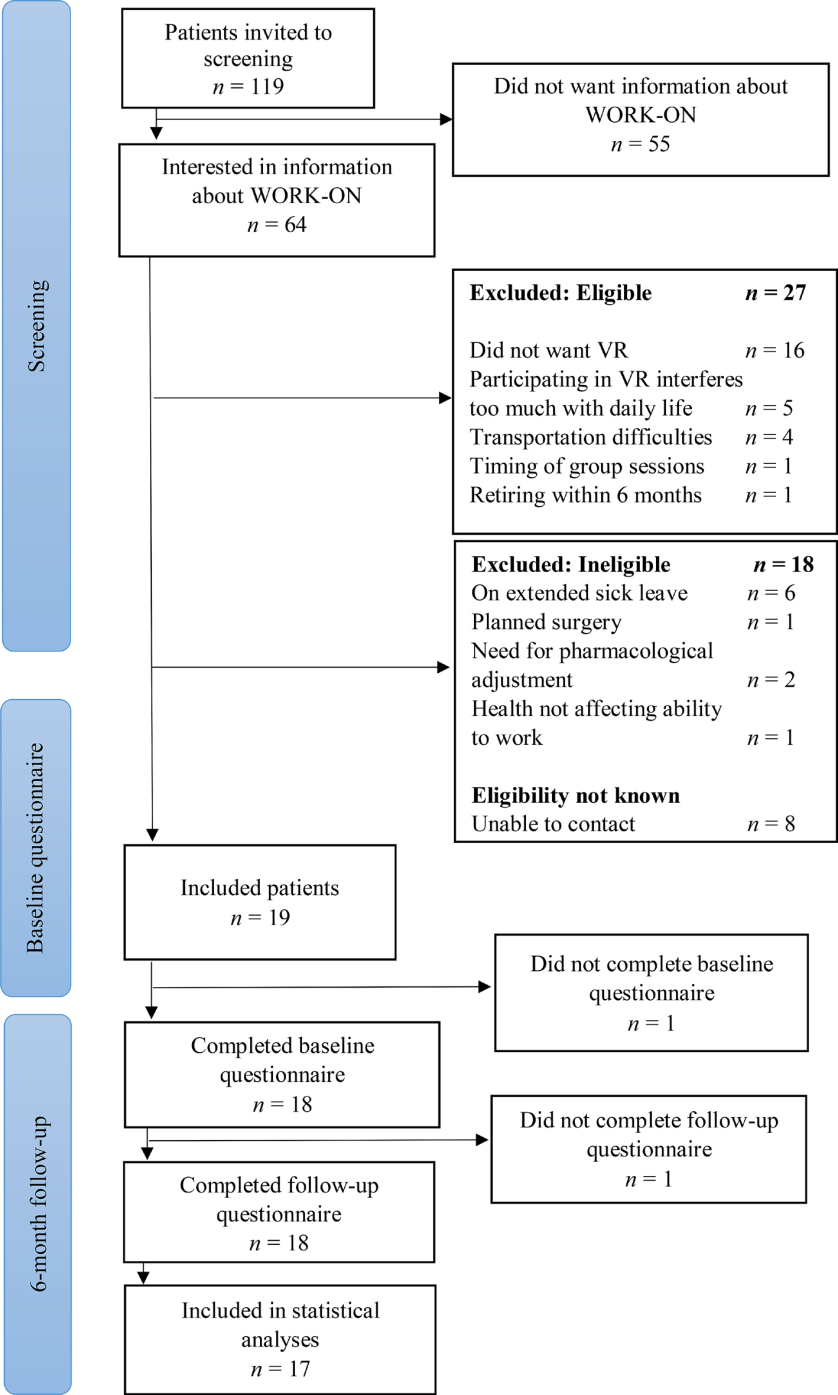
Flowchart showing the recruitment process VR, vocational rehabilitation

Between May and October 2022, 119 potentially eligible patients answered “*unlikely*” or “*not certain*” to question 6 from the WAI questionnaire [34]. Of those, 55 (46%) did not want information about WORK-ON and 64 (54%) indicated an initial interest in hearing more about the intervention. These 64 patients were contacted to assess whether they met the inclusion criteria, and those who did received more information about WORK-ON. In total, 18 of the 64 patients (28%) failed to meet the inclusion criteria and 27 patients (42%) declined to participate for various reasons (Fig. 2). Thus, 19 patients (30%) were included in the WORK-ON feasibility study.

All 19 participants completed the 6-month WORK-ON intervention. In total, 17 participants (89%) completed both the baseline and the 6-month follow-up questionnaires (Fig. 2).

### Participants

The participants were 17 women and two men aged from 34 to 64 years (Table 2). In total, 15 of the participants were diagnosed with RA, two with axSpA, and two with PsA. The median disease duration was 9 years (range, 1–18 years). Additional sociodemographic information about the participants is listed in Table 2.

**Table 2:** Sociodemographic information describing participants at baseline

Characteristic	Total
Age (years): Median [min.–max.]	55 [34–64]
Sex: *n* (%)	
Women Men	17 (89.5) 2 (10.5)
Marital status: *n* (%)	
Married / Living with partner	10 (52.6)
Living at home with children	7 (36.8)
Living alone	7 (36.8)
Diagnosis: *n* (%)	
Rheumatoid arthritis	15 (78.9)
Axial spondyloarthritis	2 (10.5)
Psoriatic arthritis	2 (10.5)
Disease duration (years): Median [min.–max.]	9 [1–18]
Employment status: *n* (%)	
Full-time	12 (63.1)
Flexi-job*	2 (10.5)
Part-time	3 (15.7)
Self-employed	3 (15.7)
Full-time education	2 (10.5)
Highest education level attained: *n* (%)	
Primary school	0
High school	2 (10.5)
Short-term further education (< 3 years)	4 (21.1)
Higher education (> 3 years)	12 (63.1)
Long-term higher education (university)	1 (5.3)
Physical activity while at work: *n* (%)	
Mostly stationary work	6 (31.6)
Mostly standing or walking	7 (36.8)
Standing or walking with lifting or carrying	6 (31.6)
Physically demanding job	0
Comorbidities: *n* (%)	
Stroke	1 (5.3)
Chronic obstructive pulmonary disease	2 (10.5)
Cancer	1 (5.3)
Osteoarthritis	6 (31.6)
Osteoporosis	2 (10.5)
Asthma	2 (10.5)
Depression	3 (15.7)

* Flexi-job is an offer in Denmark in which the municipality pays a subsidy to the employer for citizens, who have decreased work abilities and are only able to work less than half time

### Intervention delivery

Attendance and time spent in the VR are illustrated in Table 3. Time spent with the patient, documenting, and collaborating with other professionals was included in the calculations. Because of sick leave, 5 of the 19 participants were not able to attend all three group sessions and were offered individual consultations as compensation—these consultations are included in the number of individual consultations. The participants were encouraged by the coordinating OT to involve their employer. Twelve participants wanted to speak to their employers directly, without involving the coordinating OT. In these cases, the coordinating OT followed up on this part through the VR. One participant wanted to involve their employer in the VR, but the employer did not want to participate. Six of the participants involved the municipal job center, but only one participant included the coordinating OT in the contact. The participants spent an average of 13.5 h (range, 8.5–18.5) on the intervention during the 6-month study period.

**Table 3:** Resource use

***Individual consultations***	
**Healthcare professional**	**Time per participant: Hours**
Coordinating occupational therapist (OT)	10.3
Social worker	2.5
Physiotherapist	0.3
Nurse Total	2.2 15.3
***Group sessions***	
**Number**	**Participant attendance**: ***n***
1	1
2	4
3	14
***Relevant stakeholder contact***	**Participants who included the coordinating OT in the contact**: ***n***
Employer	0
Municipal job center	1
Relatives	1

### Intervention fidelity

Intervention fidelity was assessed based on journal and logbook entries. Four learning meetings with the RCs (1 h each) were held during the intervention period. These meeting times are not included in the calculations in Table 3.

Based on an initial assessment using a Danish translation of the WES-RC [33], participants identified and prioritized their occupational problems and set goals via SDM together with the coordinating OT [30]. Goals encompassed aspects such as clarification on whether the participant had the right job or wanted to become better at saying no in pressured work situations. After the initial assessments and goal setting, the participants attended group sessions. Participants were organized into one of three groups: one group had seven participants and the other two had six participants each. The RCs used the WORK-ON manual to plan and conduct the group sessions. The coordinating OT assessed the need for individual consultations over the 6-month intervention period in collaboration with each participant in accordance with the person-centered approach described in the manual.

### Outcome measures

#### Primary outcomes

For the WAI, nine participants reported an improvement in their work ability. Only 13 of the participants completed the WPAI: GH, why these results are difficult to interpret (Table 4).

**Table 4:** Change from baseline in assessed outcomes at 6-month follow-up

Variable	Baseline	Follow-up
WAI single item	(*n* = 17)	(*n* = 17)
Median [IQR]	6 [6–7]	7 [5–8]
WPAI: GH:	(*n* = 13)	(*n* = 13)
Absenteeism		
Absence from work due to ill health last week: *n* (%)	6 (46)	6 (46)
Time missed (%) because of ill health last week: Median [IQR]	0 [0–43]	0 [0–16]
Presenteeism		
Reduced productivity at work last week: *n* (%)	13 (100)	12 (92)
Percentage impairment while working due to ill health last week: Median [IQR]	50 [30–60]	50 [20–70]
Overall work impairment: Median [IQR]	71 [60–83]	70 [33–83]
Activity impairment: Median [IQR]	60 [60–70]	70 [30–80]
EQ-5D-5 L: Median [IQR]	(*n =* 17)	(*n* = 17)
Mobility	2 [2–3]	2 [2–3]
Self-care	2 [1–3]	1 [1–2]
Usual activities	3 [3–3]	2 [2–4]
Pain/Discomfort	3 [3–3]	3 [3–3]
Anxiety/Depression	2 [1–2]	1 [1–2]
EQ-VAS: Median [IQR]	(*n =* 17) 62 [43–74]	(*n =* 17) 71 [40–75]
Pain: *n* (%)	(*n =* 17)	(*n =* 17)
Little pain	0	2 (12)
Moderate pain	3 (18)	8 (47)
Strong pain	14 (82)	7 (41)
Pain that prevented working: *n* (%)	(*n =* 17)	(*n =* 15)
Little pain	1 (6)	4 (27)
Moderate pain	6 (35)	7 (47)
Strong pain	10 (59)	4 (27)
BRAF-NRSv2: Median [IQR]	(*n =* 17)	(*n =* 17)
Severity (0–10)	7 [6–8]	7 [6–8]
Impact (0–10)	7 [7–8]	7 [6–7]
Coping (0–10)	5 [5–7]	5 [3–5]
OBQ-11: Median [IQR]	(*n =* 16) 25 [17–30]	(*n =* 16) 25 [22–33]
Physical activity: *n* (%)	(*n =* 16)	(*n =* 16)
Hard exercise	0	0
Sports, heavy gardening, etc.	0	1 (6)
Walking, cycling, etc.	10 (63)	12 (75)
Seated activities	6 (37)	3 (19)
Days with min. 30 min of physical activity: Median [IQR]	(*n =* 16) 4 [1–4]	(*n =* 16) 3 [2–5]
Sitting time (hours and minutes): Median [min.–max.]	(*n =* 17)	(*n =* 17)
Transport	1 [0–2.59]	1 [0–4]
Work	3.31 [0–8.29]	5 [0–8.30]
Screen	3 [1–6]	3 [0–9]
Other	3 [0–4]	2.30 [0–5]
Sleep over the last 4 weeks: Median [min.–max.]	(*n =* 17)	(*n =* 17)
Sleep per day (hours and minutes)	6.16 [4–9]	6.30 [5–10]
Do you feel rested? *n* (%)		
Yes	1 (6)	6 (35)
No	16 (94)	11 (65)
Did you have trouble falling asleep? *n* (%)	(*n =* 16)	(*n =* 17)
Not in the last 4 weeks	3 (19)	3 (18)
1–2 times a week or less	9 (56)	10 (58)
Three or more times a week	4 (25)	4 (24)
Did you wake up several times at night and have difficulty		
falling asleep? *n* (%)	(*n =* 17)	(*n =* 17)
Not in the last 4 weeks	2 (12)	3 (18)
1–2 times a week or less	6 (35)	6 (35)
Three or more times a week	9 (53)	8 (47)
Did you wake up early and were unable to fall asleep		
again? *n* (%)	(*n =* 17)	(*n =* 17)
Not in the last 4 weeks	4 (24)	4 (24)
1– 2 times a week or less	6 (35)	8 (47)
Three or more times a week	7 (41)	5 (29)
Did you sleep uneasily? *n* (%)	(*n =* 16)	(*n =* 17)
Not in the last 4 weeks	1 (6)	2 (12)
1–2 times a week or less	8 (50)	7 (41)
Three or more times a week	7 (44)	8 (47)
WHO-5 Well-being Index: Median [IQR]	(*n =* 17) 18 [15–23]	(*n =* 17) 17 [12–21]

BRAF-NRSv2, Bristol Rheumatoid Arthritis Fatigue Numerical Rating Scales version 2; EQ-5D-5 L, European Quality of Life—5 Levels; EQ-VAS, EuroQol Visual Analogue Scale; IQR, interquartile range; OBQ-11, Occupational Balance Questionnaire; WPAI: GH, Work Productivity and Activity Impairment Questionnaire: General Health; WAI, Work Ability Index; WHO, World Health Organization

Because the sample size was small, the study was not powered to show significant changes in the outcome measures.

### Sick leave

The 19 participants reported an average of 2.8 days (range, 0–112 days; median, 10 days) of sick leave measured by text messages per month. In addition, several participants revealed in their text messages that they felt they should have taken days of from work because of the impact of their IA but did not.

**Fig. 3:**
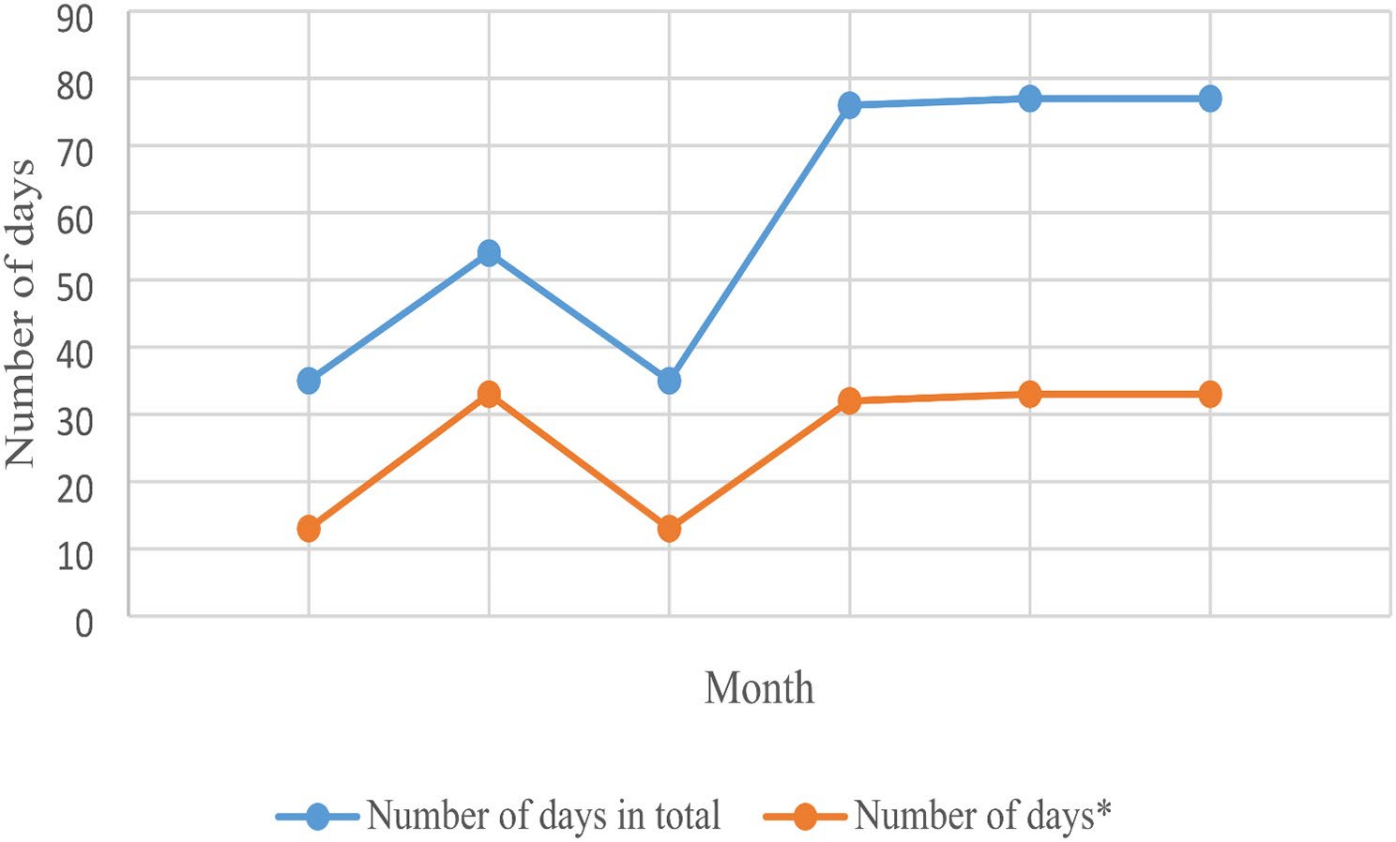
Days of sick leave recorded for each month throughout the intervention (*n* = 19) Number of days* = number of days without two participants on long-term sick leave throughout the vocational rehabilitation

One participant went on full-time sick leave immediately after inclusion in WORK-ON and accumulated 112 days of sick leave during the intervention period. Another participant went on full-time sick leave in the final 3 months of the intervention, leading to an increase in the total number of days of sick leave during months 4–6, as shown in Fig. 3. Further, employees in part-time work had more days of sick leave than those in full-time work. Participants working full-time had an average 1 day of sick leave (median, 4 days) per month and those working part-time had an average 5.8 days of sick leave (median, 23 days) per month. Both the participants who went on full-time sick leave worked part-time.

## Discussion

The study reported here evaluated the feasibility of WORK-ON, with a focus on recruitment procedure, intervention fidelity and delivery, feasibility of outcome measures, and evaluation of which outcome measure to use as the primary outcome measure in a subsequent RCT. The findings from this study are from a Danish context, but can be used in VR in other countries. Overall, the study demonstrated WORK-ON is feasible and suitable for people with IA. However, in the recruitment process, a majority (69%) of the patients who had indicated they wanted to hear more about the study following the initial screening either did not want information about WORK-ON (*n* = 55) or declined to participate in the intervention for various reasons (*n* = 27). WORK-ON is a complex intervention addressing several issues, and involvement requires active engagement from the participant. Some of the patients who declined participation may have perceived the intervention as too comprehensive and interfering too much with their work and daily life. Another possible challenge patients may have perceived is the need to balance work with other occupations in everyday life, potentially resulting in a lack of energy to participate in WORK-ON.

Further, 13 of the 19 participants had a higher level of education and only two were men. This raises the question of whether the intervention primarily targets women with a higher social status and, consequently, leads to the exclusion of eligible patients with a high need for VR. Therefore, we must consider whether the current version of WORK-ON contributes to social inequality and, if so, how to avoid this. Research indicates social factors such as low education, low income, and unemployment are closely linked to health [45]. Moreover, higher wages and educational levels are associated with a reduced risk of long-term sick leave and early retirement [46]. In the recruitment process, possible participants were shown a pop-up text with a short text about the study after completion of a battery of questions in DANBIO. If some patients found reading and understanding this text difficult because of low health literacy [47], this could be a potential barrier to participation. Further, the text contained information about how many hours and consultations participants might spend in the intervention, which may have discouraged some from hearing more about the study and participating. Thus, adjustment of the pop-up text may be necessary. In a future RCT, recruitment can also involve the clinical staff of rheumatologists and nurses in the outpatient clinic, as they can introduce WORK-ON during consultations with eligible patients. This approach may enable inclusion of eligible patients.

In the individual consultations, participants consulted the social worker more than the other RCs. Journal entries revealed participants needed to discuss their options for economic support and compensatory schemes because of their employment situations. The physiotherapist and the nurse were consulted less frequently than expected. In the feasibility study, the physiotherapist was not present in the group sessions; therefore, participants may have been less likely to ask for a physiotherapy consultation if they did not have a clear understanding of what the physiotherapist could offer. There might not have been enough focus on how a physiotherapist could support participants in physical activity or the importance of physical activity, which should be in focus in a future study. However, the nurse was present in the group sessions but only three participants asked for a nursing consultation. As part of their standard care in the outpatient department, the participants already had regular appointments with a rheumatology nurse. They may not have understood that, in WORK-ON, the nurses did not have to spend time assessing blood tests and responses to questionnaires or performing joint assessments as in their usual nursing consultations, and thus there were greater opportunities for support to understand and manage (i.e., the pain and fatigue) their disease. There is a need for more focus on fatigue in a subsequent RCT, as fatigue is one of the main challenges for people with IA in work [16].

Both the WAI single item and WPAI: GH have been recommended for measuring at-work productivity loss (presenteeism), which is a major problem in people with IA [7, 11, 48]. The WAI single item has been shown to be a reliable short substitute for the more extensive WAI and to have acceptable convergent validity [49]. Still, the WAI single item only refers to the participants’ current work ability on a scale from 0 to 10 [49]. The WAI single item has been criticized for not being able to identify the risk of disability pension or long-term sick leave, and being too simple for not allowing specific determinants of work ability to be identified, although the short form of the WAI single item is simple to interpret and may increase completion rates [50].

The WPAI: GH evaluates four outcomes: absenteeism, presenteeism, overall work impairment, and activity impairment [37]. In the feasibility test, we obtained slightly different results from the WPAI: GH in relation to work productivity and overall work impairment than other studies with similar target groups [51–53]. This may be because the few participants in the feasibility test had lower levels of work productivity and higher degrees of overall work impairment than the participants in the other studies [51–53]. This could be due to the recruitment process or the small sample size in present study. Notably, only 13 out of 19 participants completed the WPAI: GH. The cognitive interviews we undertook with three patients prior to the feasibility test indicated no problems in understanding or completing the WAI single item or the WPAI: GH, but the three patients may not be representative of the study population. Another study found the formulation of the questions in WPAI: GH and the word “productivity” may be difficult to understand if participants’ work does not involve “production of products” [48]. To ensure a higher response rate from the WPAI: GH, when developing the REDCap questionnaire, a future RCT should ensure all questions are marked as “required” so that participants cannot proceed with the questionnaire until the question is answered. Therefore, the WPAI: GH may not considered suitable as a primary outcome measure for a subsequent RCT. Work ability is important, as it may serve as an indicator for future long-term sick leave, early retirement, and job loss [46]. While job loss could be considered a potential primary outcome, a power calculation suggests recruiting sufficient participants within a reasonable timeframe and a long follow-up may be challenging, as reduction of job loss will probably be a long-term outcome in accordance with our logic model for the association between job loss and work ability [26]. The WAI single item can be considered a proxy for subsequent job loss, and it is a simple question to answer [34]. As there is no validated Danish version of the full WAI questionnaire, we may choose the WAI single item as the primary outcome measure in a subsequent RCT.

An unintended consequence we identified through the feasibility study was an increase in sick leave, and one of the aims of WORK-ON was to decrease sick leave. When assessing intervention fidelity, we found the coordinating OTs encouraged the participants to take more sick leave to increase their ability to self-manage their lives, improve their occupational balance, and enhance their capacity to continue paid work. This may lead to decreased presenteeism at work, but it was a key uncertainty we did not anticipate from the beginning. Some participants had a compensatory scheme (a legal agreement) with the municipality (called a “§ 56” in Denmark) where they can take a minimum of 10 days of sick leave because of their IA, and the employer receives economic compensation from the municipality. Often, patients do not use this agreement in consideration of their colleagues and employer, but they will lose the § 56 if they do not use it at least 10 days per year. Therefore, participants may have been encouraged to increase their sick leave days after starting the intervention. Overall, increased sick leave is unacceptable from a socio-economic perspective. This calls for a discussion of the need for additional adjustments and support as part of WORK-ON to enable participants to manage their work. Further, given the feasibility study was not a randomized design, we lack the ability to discern whether sick leave differed from a control group. According to Statistics Denmark, in 2021, men had 6.9 days of sickness absenteeism per year and women 11.8 days on average [54]. A Danish report from 2017 mentions that people with RA had 5.6 more days away from work because of illness than the general population in Denmark, which aligns with our findings [55]. People with IA experience a mean productivity loss of 23.5% each week in 2020 according to a small Danish study [53]. Likewise, a report from the University of Southern Denmark in 2022 showed sick leave was more frequent among people with musculoskeletal disorders (23.4%) than among people without them (16.5%) [56]. Further, in present study there seemed a tendency to more sick leave among participants in part-time work compared with participants in full-time work. Those in part-time work may already have been through a process of accepting their disease and managed their condition by working fewer hours.

None of the participants wanted to involve the coordinating OT in contact with their employer. This is in line with another feasibility study testing job retention for people with IA in the United Kingdom, which found few participants wanted to involve their employer [57].

Finally, we developed a logic model for WORK-ON [26] and found several of our assumptions were confirmed. For example, the results indicated improvements in quality of life, physical activity, and pain reduction. These improvements could be due to increased focus on energy management and coping strategies, decreasing the impact of IA. However, the logic model did not show improvements in outcome measures for occupational balance and mental well-being as we anticipated [26].

### Strengths and limitations

A major strength of our WORK-ON study was that it was carried out at the DHRD, which has specialized resources for rehabilitating people with rheumatic diseases and access to RCs who practice a person-centered approach, are skilled in FACT and goal setting, which are key elements of WORK-ON. Therefore, the RCs have expertise in the biopsychosocial rehabilitation of people with IA. Importantly, this feasibility study was not intended or powered to find statistically significant differences in the two primary outcomes tested. A limitation was the missing information for two of the participants in the outcomes measures, and six of the participants did not complete the WPAI: GH. Another limitation was no employers were involved directly. Employers are important stakeholders in VR, and evidence shows employers want involvement in VR and are prepared to adjust work activities and arrange flexible working hours for employees [27]. A subsequent RCT should place more emphasis on involving employers to ensure VR is successful. However, respecting a participant’s decision not to involve their employer is also important. A third limitation is that we did not include observations as part of the assessment of intervention fidelity but only journal and logbook entries. Notably, some participants reported comorbidities not identified during eligibility screening (e.g., stroke, cancer, and depression). Because these data were self-reported, whether these comorbidities had an impact on the participants’ work ability is uncertain. In addition, one eligible patient planned to retire within six months. Our eligibility criteria and recruitment should be refined to take these issues into account for a subsequent RCT.

## Conclusion

The WORK-ON intervention is considered feasible, although some adjustments are needed before testing in a subsequent RCT. Recruitment should be adjusted to include rheumatologists and nurses in the outpatient clinic. In general, there is a need to focus more on the use of FACT and fatigue management. Further, a focus on how to prevent sick leave by adjusting participants’ work environment and employer involvement is necessary. Finally, the logic model needs to be modified to describe what happens and what works for whom, and further training of the RCs in fatigue needs to be planned and delivered.

We believe an adjusted version of WORK-ON has the potential to support patients with IA to increase their work ability, resulting in a reduction in job loss. The WAI single item may be used as the primary outcome and job loss as a secondary outcome in a subsequent RCT. A full evaluation of costs in relation to outcomes is planned as part of a subsequent RCT.

## Data Availability

The datasets used and/or analyzed during the current study are available from the corresponding author on reasonable request.

## Abbreviations

AxSpA: Axial spondyloarthritis
BRAF-NRSv2: Bristol Rheumatoid Arthritis Fatigue Numeric Rating Scales version 2
CONSORT: Consolidated Standards of Reporting Trials
DHRD: Danish Hospital for Rheumatic Diseases
EQ-5D-5L: European Quality of Life—5 Levels
EQ-VAS: EuroQol Visual Analogue Scale
FACT: Focused acceptance and commitment therapy
IA: Inflammatory arthritis
IQR: Interquartile range
MRC: Medical Research Council
OBQ-11: Occupational Balance Questionnaire
OT: Occupational therapist
PsA: Psoriatic arthritis
RA: Rheumatoid arthritis
RCT: Randomized controlled trial
REDCap: Research Electronic Data Capture
VR: Vocational rehabilitation
WAI: Work Ability Index
WES-RC: Work Experience Survey—Rheumatic Conditions
WHO: World Health Organization
WPAI: Work Productivity and Activity Impairment Questionnaire: General Health
